# Stress cardiomyopathy as a cardiovascular manifestation of COVID-19

**DOI:** 10.21542/gcsp.2023.29

**Published:** 2023-09-30

**Authors:** Khaled M. Abdullah, Mohamad Bakir, Danielle Greenberg, Eyad Almasri

**Affiliations:** 1 UCSF Fresno, San Francisco, USA; 2 Alfaisal University, College of Medicine, Riyadh, Saudi Arabia; 3 UCSF Fresno, Pulmonary/Critical Care, San Francisco, USA

## Abstract

Patients with coronavirus disease 2019 (COVID-19) may present with a broad spectrum of clinical manifestations, affecting several organ systems. Predominant cardiac manifestations include myocardial injury, heart failure, cardiogenic shock, and arrhythmias. Stress (takotsubo) cardiomyopathy, characterized by apical ballooning of the heart leading to acute left ventricular dysfunction, is rarely seen in patients with COVID-19. We present a case of COVID-19-associated stress cardiomyopathy in a female in her sixties.

## Background

Takotsubo cardiomyopathy, also known as stress cardiomyopathy (SCM), is a type of non-ischemic cardiomyopathy that primarily affects postmenopausal women^1^. The condition is distinguished by temporary left ventricular regional systolic dysfunction in the absence of abrupt plaque rupture or angiographically severe coronary artery disease^1^. The manifestation of takotsubo cardiomyopathy is comparable to that of acute coronary syndrome. The most prevalent symptoms in the International Takotsubo Registry are chest pain, dyspnea, and syncope. Some patients may have signs and symptoms of heart failure, arrhythmias, sudden cardiac arrest, or significant mitral regurgitation^1^.

## Case presentation

Our patient was a female in her sixties with a past medical history of hypertension, hyperlipidemia, and COPD, who presented to the emergency department with a one-day history of sudden-onset chest pain. She described it as sharp, non-radiating, non-positional, substernal, and intermittent, with each episode lasting approximately 3 min with no exacerbating or relieving factors.

The pain was associated with sudden and progressively worsening shortness of breath. She denied any orthopnea, lower leg swelling, fevers, chills, or cough. She noted that she was in contact with her mother who was recently diagnosed with COVID-19. She received two doses of the COVID vaccine with the last dose approximately 8 months prior to admission. She also noted that she was diagnosed with COVID-19 three months prior to this admission and was never admitted to the ICU before.

Vitals were significant for tachycardia; up to 124 beats per minute, tachypnea of 31 breaths per minute, and pulse oximetry of 78% which increased to 95% on high flow nasal cannula (55 liters per minute with an O_2_ fraction (FiO_2_) of 60%). On examination, the patient was mildly overweight and in acute respiratory distress using accessory respiratory muscles. Jugular venous distension up to the mid-neck was noted, bibasilar crackles were auscultated, and trace edema was noted in the lower extremities. Otherwise, the exam was unremarkable including cardiac auscultation.

**Figure 1.**
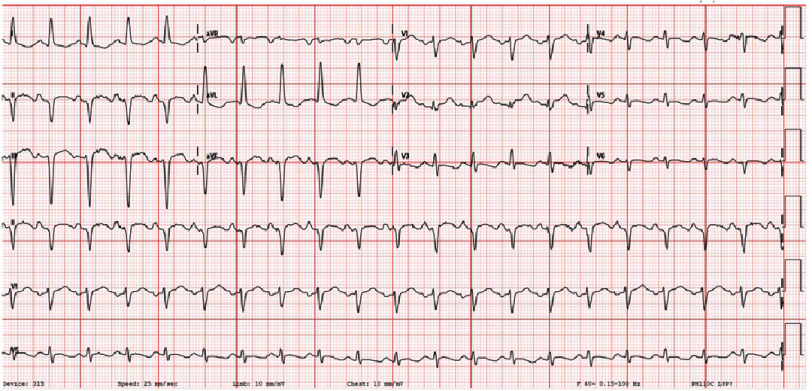
EKG on admission demonstrating sinus tachycardia and nonspecific ST changes.

**Figure 2.**
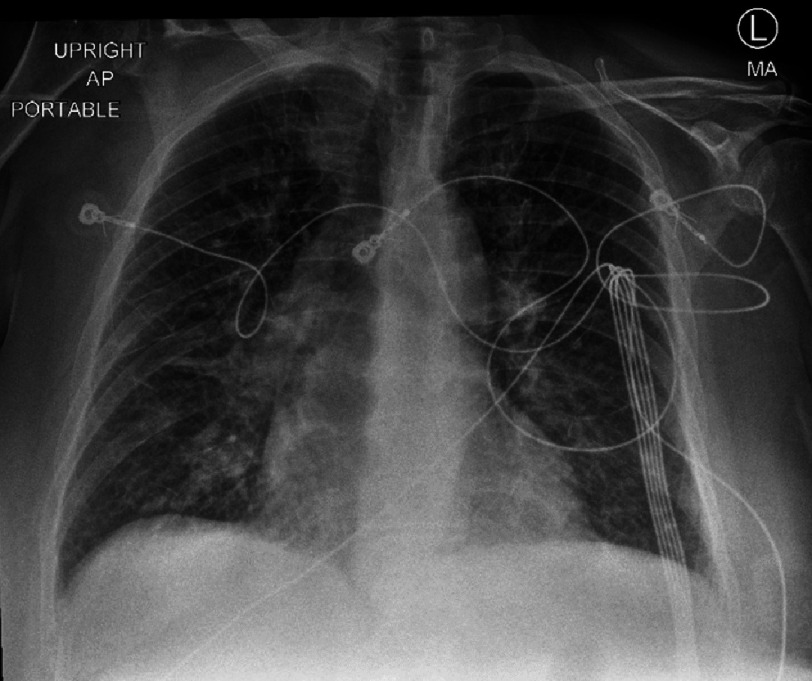
Chest X-ray on admission showing diffuse interstitial opacities.

Initial lab results (Table 1) showed a normal complete blood count but complete metabolic panel significant for mild hypokalemia and an elevated AST. The patient was found to be positive for COVID-19 by PCR with a negative viral and bacterial respiratory pathogen panel. EKG (Figure 1) on admission was significant for sinus tachycardia and ST changes. Chest X-ray (Figure 2) showed diffuse interstitial opacities. Troponin was elevated at 5.7. BNP was elevated to 205.

**Table 1:** Laboratory test results.

Lab test (unit)	Results
WBC Count (K/uL)	10.2
Hemoglobin (g/L)	15.4
Platelets (×10^9^/L)	202
Sodium (mmol/L)	141
Potassium (mmol/L)	3.3 ↓
Chloride (mmol/L)	106
Bicarbonate (mmol/L)	19
BUN (mmol/L)	17
Creatinine (mmol/L)	0.6
AST (mmol/L)	136 ↑
ALT (mmol/L)	11

**Notes.**

WBC White Blood Cell BUN Blood Urea Nitrogen AST aspartate aminotransferase AST alanine aminotransferase

**Figure 3.**
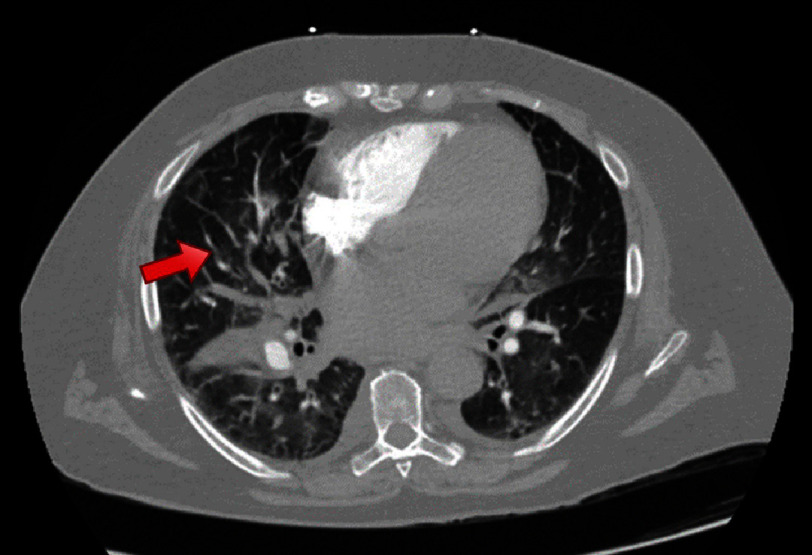
CT showing segmental pulmonary embolism (red arrow) within the anterior pulmonary artery branch to the right upper lobe.

The initial provisional diagnosis was a pulmonary embolism (PE). Computerized tomography of the chest with angiography showed diffusely spread ground glass opacities, interlobular thickening, and a small segmental PE within the anterior pulmonary artery branch to the right upper lobe (Figure 3). However, the troponin elevation could not be solely explained by a PE. Moreover, there were no findings suggestive of right heart strain on the EKG or CT. In the setting of chest pain, EKG findings concerning for ischemia, and a significant troponin elevation, myocardial infarction was the most life-threatening diagnosis that must be ruled out. Other noteworthy differentials included myocarditis, SCM, and less likely COPD exacerbation.

**Figure 4.**
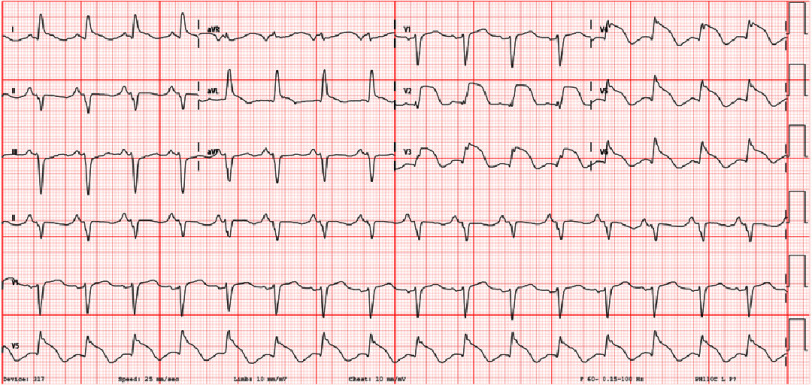
EKG demonstrating convex ST elevations in leads v2–v6.

Across the next 24 h, BNP increased to 2333, and troponins continued to rise and peaked at 32. The patient complained of worsening chest pain the following evening, and an EKG (Figure 4) obtained at that time showed significant convex ST elevations from V2 to V4 with no reciprocal changes in the inferior leads. Thus, urgent catheterization was done showing no angiographic evidence of coronary artery disease but an estimated ejection fraction (EF) of 10–15%. The most likely differential diagnosis at this point was SCM, but definitive imaging was required.

An echocardiogram demonstrated global hypokinesis. However, due to poor acoustic windows, it was unclear if there was markedly worsened wall motion near the apex of the left ventricle. A cardiac MRI (Video 1 & 2) demonstrated a hyperdynamic basal segment with severely hypokinetic mid to apical ventricle with overall findings that are suggestive of atypical takotsubo cardiomyopathy given classic apical ballooning appearance.

Initially, a heparin infusion was started in addition to aspirin and clopidogrel due to concern for non-ST elevation myocardial infarction in addition to overlapping therapy for a pulmonary embolism. After coronary angiography, 40 mg of intravenous furosemide twice a day was started. The patient symptomatically improved with diuresis. Her oxygen requirements decreased, and the patient was comfortable on 3 liters nasal cannula with an oxygen saturation of 95%.

Once out of acute heart failure exacerbation secondary to SCM, the patient was started on goal-directed medical therapy with the initiation of metoprolol tartrate and losartan throughout the hospital course and transitioned to metoprolol succinate prior to discharge. The patient was weaned off oxygen therapy and was comfortably saturating more than 95%. She was discharged with close follow up in the heart failure clinic. She regularly follows up and was not complaining of any symptoms during her time in the clinic.

**Video 1. Cardiac MRI (4 chamber view).** Demonstrating hyperdynamic basal segment with severely hypokinetic mid to apical ventricle. https://drive.google.com/file/d/1bMAAwO9594ktwZaC829KotKmryLlFaae/view?usp=share_link

**Video 2. Cardiac MRI (2 chamber view).** Demonstrating hyperdynamic basal segment with severely hypokinetic mid to apical ventricle. https://drive.google.com/file/d/1aY7PFqcPaS4uFsmijl4SNibuuT2LFON3/view?usp=share_link

## Discussion

The most widely accepted diagnostic criteria for stress cardiomyopathy is the Mayo Clinic Criteria. The following criteria must all be present in order to meet the diagnosis: Transient hypokinesis, akinesis, or dyskinesis in the left ventricular mid segments with or without apical involvement; regional wall motion abnormalities extending beyond a single epicardial vascular distribution; and, more often than not, a stressful trigger; new ECG abnormalities (T-wave inversion and/or ST-segment elevation); no obstructive coronary disease or angiographic evidence of acute plaque rupture; and no myocarditis or pheochromocytoma^2,3^.

There is no clear understanding of the exact etiology of SCM. Several theories, including sympathetic overdrive with elevated catecholamines, microvascular dysfunction, coronary spasm, inflammation, low estrogen levels, or defective myocardial fatty acid metabolism, have been proposed as potential etiologies of SCM^4,5^. According to one study, patients with COVID-19 have much greater levels of cortisol than those following major surgery^6^. It is probable that excessive concentrations of cortisol, along with elevated levels of catecholamines, have a direct toxic effect on cardiomyocytes in COVID-19 patients, contributing to the development of SCM^7^. Moreover, COVID-19’s procoagulant condition and proinflammatory cytokine storm raises the risk of heart injury, coronary vasospasm, sudden myocardial infarction and shock^8^.

The initial focus of management should be on identifying and closely monitoring patients who are at risk of serious complications. Because there is no prospective randomized evidence on the management of SCM, care is primarily based on clinical experience and expert opinion. Since it initially presents as potential acute coronary syndrome, the initial treatment includes aspirin, an ACE inhibitor, beta-blockers, a lipid-lowering drug, and coronary angiography to clear out obstructive coronary artery disease^9^. ACE inhibitors and cardioselective beta-blockers are used for a brief period of 3–6 months in stable patients, with serial imaging scans to evaluate ventricular ejection fraction and wall motion abnormalities to identify deterioration or recovery. Anticoagulation is often reserved for individuals with established evidence of embolic events or ventricular thrombus. Inotropes must be administered to patients who present in cardiogenic shock in the absence of left ventricular outflow obstruction or who have more unstable hemodynamics^1^.

Even though most patients with COVID-19-induced SCM improve within weeks or months, the case fatality rate in COVID-19-induced SCM is as high as 36.5%. This is much greater than in SCM patients who do not have COVID-19, which varies from 0.95−2.3% in those who do not have cardiogenic shock to 13.6–23.5% in those who do have cardiogenic shock^10,11^. Likewise, SCM appears to be linked with worse outcomes in COVID-19 patients. Cases of COVID-19 worsened by SCM often had a more severe illness than those without^10^.

### Learning points

• COVID can have several cardiac manifestations including arrhythmias, myocarditis, hypoxic myocardial injury, and SCM.
• A broad spectrum of clinical presentations exists from asymptomatic to florid heart failure exacerbation.
• Cardiac troponin and natriuretic peptide biomarkers are commonly elevated among patients with COVID-19 and are associated with increased mortality rate.
• Echocardiography findings include right ventricular dilation and dysfunction as well as left ventricular systolic and diastolic dysfunction.
